# Coronary Revascularization in the Era of TAVR: Timing, Strategy, and Outcomes

**DOI:** 10.1177/11795468251395832

**Published:** 2025-12-01

**Authors:** Avery Love, Chandler O’Leary, Shahman Shahab

**Affiliations:** 1 The University of Texas Medical Branch, Galveston, USA

**Keywords:** TAVR, PCI timing, coronary artery disease, revascularization, interventional cardiology

## Abstract

**Background:**

Coronary artery disease (CAD) is prevalent among patients undergoing transcatheter aortic valve replacement (TAVR), complicating clinical decision-making regarding optimal timing of percutaneous coronary intervention (PCI). Despite widespread clinical experience, there is ongoing controversy and limited consensus regarding when PCI, before, during, or after TAVR, offers the best risk-benefit balance.

**Objective:**

To synthesize and critically evaluate the current evidence on different PCI timing strategies in patients undergoing TAVR and to identify areas of uncertainty and clinical complexity.

**Review:**

Randomized trials, including ACTIVATION and NOTION-3, have yielded mixed findings, highlighting a modest reduction in ischemic events with pre-TAVR PCI but increased procedural bleeding risks. Observational registries (REVASC-TAVI and the National Readmissions Database) have similarly shown that pre- and peri-TAVR PCI strategies carry heightened risks of complications without clear long-term mortality benefits. Recent meta-analyses reinforce these findings, indicating that no PCI timing strategy conclusively outperforms others in reducing long-term mortality. Key considerations such as anatomical complexity, frailty, bleeding risk, and valve type significantly influence optimal PCI timing.

**Conclusion:**

Current evidence supports a personalized, patient-centered approach to PCI timing in TAVR candidates, emphasizing careful consideration of individual anatomical and clinical factors rather than a standardized timing protocol. Further research exploring advanced risk stratification, imaging modalities, and long-term clinical outcomes is essential to refine current guidelines and practice.

## Introduction

Aortic stenosis (AS) and coronary artery disease (CAD) frequently coexist due to shared risk factors such as advanced age, hypertension, and atherosclerosis.^1,2^ As transcatheter aortic valve replacement (TAVR) continues to expand across all surgical risk categories, clinicians are increasingly confronted with the challenge of managing CAD in patients undergoing TAVR. Current data suggest that approximately 40% to 75% of patients referred for TAVR have significant obstructive CAD, raising important questions about the timing, necessity, and approach to revascularization in this population.^3^

The management of CAD in TAVR candidates presents unique challenges. Untreated coronary lesions may compromise procedural safety or long-term outcomes, while PCI in the setting of severe AS carries its own risks including bleeding, contrast-induced nephropathy, and hemodynamic instability.^4^ Moreover, the valve type and implantation depth used during TAVR can significantly affect future coronary access, making the timing of PCI a critical consideration.^5,6^

Clinical practice currently varies widely, with some centers performing PCI before TAVR to optimize coronary perfusion, while others defer PCI until after valve implantation or perform both procedures concomitantly.^7^ Randomized evidence remains limited. The ACTIVATION trial, a rare RCT in this domain, did not demonstrate clear benefit of PCI before TAVR, and observational studies have yielded conflicting results.^8^ As a consequence, both U.S. and European guidelines provide only general recommendations, emphasizing individualized treatment strategies without defining an optimal approach.

This review aims to synthesize the current evidence on PCI timing in patients undergoing TAVR. We reviewed relevant randomized trials, registries, and meta-analyses published between 2010 and 2025, prioritizing high-impact studies and guideline-influencing evidence. Editorials and studies without outcome data were excluded from consideration. We explore the advantages and limitations of pre-, peri-, and post-TAVR PCI strategies, analyze key studies and guideline statements, and highlight practical considerations for coronary access, procedural planning, and individualized care. In doing so, we aim to provide a contemporary framework for decision-making in an area of cardiology where evidence continues to evolve.

## Current Guidelines and Practice Patterns

Current management guidelines from the American College of Cardiology/American Heart Association (ACC/AHA) and European Society of Cardiology/European Association for Cardio-Thoracic Surgery (ESC/EACTS) offer limited and general recommendations regarding the timing of PCI relative to transcatheter aortic valve replacement. Both guidelines advocate individualized decision-making based upon patient-specific anatomical considerations, clinical presentation, and procedural risk assessment, underscoring the complexity of ongoing uncertainty surrounding optimal timing strategies.^9-11^

According to the 2020 ACC/AHA guidelines, coronary revascularization in patients undergoing TAVR should be considered primarily in those with significant proximal coronary lesions, especially involving the left main coronary artery or proximal left anterior descending artery.^10^ However, the guidelines stop short of providing explicit recommendations on timing, suggesting only that decisions be individualized through multidisciplinary heart team discussions, considering lesion complexity and anticipated ease or difficulty of coronary access post-TAVR.

The 2025 ESC/EACTS guidelines similarly emphasize a tailored approach. They suggest that coronary lesions causing demonstrable ischemia or limiting coronary flow reserve should be considered for PCI before TAVR if deemed likely to impact outcomes during or after valve replacement.^12^ Nevertheless, like their American counterparts, these guidelines lack precise recommendations regarding optimal timing, reflecting the absence of robust randomized evidence.

Real-world practice patterns consequently vary significantly across institutions and geographic regions. Many centers routinely perform coronary angiography and PCI prior to TAVR, favoring the logic of optimizing myocardial perfusion and simplifying procedural logistics during valve deployment.^4^ Other centers prefer performing PCI concurrently with TAVR for efficiency, utilizing single vascular access sites, though this approach can increase procedural complexity and bleeding risk.^7,13^ Conversely, a growing number of institutions choose to defer PCI until after TAVR implantation, reasoning that valve deployment may improve symptoms sufficiently, reducing the need for immediate coronary intervention. This strategy, however, presents challenges due to compromised coronary access after valve placement, particularly with self-expanding valves or anatomically difficult implantation.^14-16^

Given the lack of definitive guidance and clear consensus, clinical decision-making remains highly individualized, emphasizing a heart team-based approach. Variability in practice patterns highlights the urgent need for further high-quality clinical trials and consensus documents to clarify optimal revascularization strategies for patients undergoing TAVR.

## Review of Key Studies

The optimal timing for PCI in patients undergoing TAVR has been investigated in various observational studies, registries, and randomized controlled trials. In this section, we review major studies that shape current clinical practice and provide a foundation for the ongoing debate regarding the best PCI timing strategy. Key findings of these studies are highlighted in Table 1.

**Table 1.** Summary of Major Studies Evaluating the Timing of Percutaneous Coronary Intervention (PCI) in Relation to Transcatheter Aortic Valve Replacement (TAVR).

#	Study name	Type	Key finding	Journal/Source
1	ACTIVATION Trial	Randomized Controlled Trial	Compared PCI prior to TAVR vs. no PCI in patients with stable CAD. At 1 y, there was no significant difference in the composite endpoint of death or rehospitalization (41.5% in PCI group vs. 44.0% in no-PCI group; *P* = .067). Major bleeding (any bleed) was significantly higher in the PCI group (44.5% vs 28.4%; *P* = .021), indicating increased procedural risk without clinical benefit in primary outcomes.	JACC: Cardiovascular Interventions (2021)
2	NOTION-3 Trial	Randomized Controlled Trial	Demonstrated a modest reduction in MACE at 1 y in patients undergoing PCI prior to TAVR compared to TAVR alone (HR 0.71; 95% CI: 0.51-0.99; *P* = .04). Rates of myocardial infarction and urgent revascularization were also significantly lower in the PCI group, though this benefit came at the cost of increased bleeding complications (HR 1.51; 95% CI: 1.03-2.22).	NEJM (2024)
3	Rheud et al (REVASC-TAVI Registry)	Multicenter observational registry study	Compared PCI timing in TAVR patients: pre-PCI (n = 1052), simultaneous (n = 394), and post-PCI (n = 157). At 2 y, all-cause mortality was lowest in the post-TAVR PCI group (6.8%) vs. pre- (20.1%) and simultaneous PCI (20.6%; *P* < .001). The composite endpoint (death, stroke, MI, HF hospitalization) was also lower post-TAVR (17.4% vs 30.4% pre-PCI and 30.0% simultaneous; *P* = .003). Findings were consistent after IPTW and landmark analysis, favoring deferred PCI.	EuroIntervention (2023)
4	Park et al (National Readmissions Database)	Observational Database Study	5207 patients, same-day PCI and TAVR (27.1%) was associated with lower rates of complications compared to PCI staged ⩽ 30 d before TAVR (41.5%) or >30 d (31.3%). Early-staged PCI was linked to higher odds of acute kidney injury (AOR 1.49; *P* = .024), non-home discharge (AOR 1.53; *P* = .001), and 90-d readmission (AOR 1.35; *P* = .026), with no difference in in-hospital mortality between groups.	Current Problems in Cardiology (2024)
5	Fallahtafti et al	Network Meta-Analysis	PCI performed during TAVR was associated with significantly higher 30-day all-cause mortality (RR 2.46; 95% CI: 1.40-4.32) and in-hospital mortality (RR 1.70; 95% CI: 1.08-2.69) compared to no PCI. It also increased the risk of 30-day myocardial infarction (RR 3.63; 95% CI: 1.27-10.43). PCI after TAVR was linked to elevated 1-year mortality relative to other strategies. There were no significant differences in major bleeding or stroke across timing strategies.	Clinical Cardiology (2024)
6	Caminiti et al	Meta-Analysis	In 1531 patients, pre-TAVR PCI was associated with higher all-cause mortality compared to deferred PCI (OR 2.48; 95% CI: 1.19-5.20; *P* = .02). No significant differences were observed in stroke (OR 3.58; *P* = .12) or MI (OR 0.66; *P* = .29) between PCI timing groups. These results support post-TAVR PCI as potentially safer in stable CAD patients undergoing TAVR	Journal of Clinical Medicine (2024)

This table highlights key findings from randomized trials, multicenter registries, observational database studies, and meta-analyses comparing pre-, peri-, and post-TAVR PCI strategies, emphasizing differences in mortality, ischemic, and bleeding outcomes across study designs and patient populations.

### Randomized Controlled Trials (RCTs)

The ACTIVATION trial (2021) was a landmark randomized study evaluating outcomes of PCI performed prior to TAVR compared to no PCI in patients with significant CAD undergoing TAVR. ACTIVATION enrolled 235 patients randomized to PCI before TAVR versus conservative management. At 1 year, the trial reported no significant difference in all-cause mortality and rehospitalization rates (noninferiority was not met, difference: −2.5%; 1-sided upper 95% confidence limit: 8.5%; 1-sided noninferiority test *P* = .067), although PCI prior to TAVR was associated with increased bleeding events (HR: 1.46; 95% CI: 0.93-2.29. *P* = .098).^8^ The results of ACTIVATION highlighted the absence of a clear benefit from routine pre-TAVR PCI, while also noting limitations in sample size and power.

The NOTION-3 trial (2024) offered additional insight. This randomized study compared PCI performed prior to TAVR versus medical therapy alone for stable CAD lesions. NOTION-3 demonstrated a modest reduction in major adverse cardiac events (MACE) in the PCI group at 1 year (HR: 0.71; 95% CI: 0.51-0.99; *P* = .04), though this came at the cost of increased procedural complications and bleeding risks (HR: 1.51; 95% CI: 1.03-2.22).^17^ This study underscored the complexity of balancing the benefits and risks associated with pre-TAVR PCI.

### Observational Studies and Registries

The REVASC-TAVI registry provided real-world data from multiple European centers evaluating outcomes based on PCI timing. The study revealed significant heterogeneity in practice, with approximately 66% of patients undergoing PCI before TAVR, 25% concomitantly, and 10% after TAVR. Importantly, the registry demonstrated that PCI performed after TAVR was associated with significantly lower 2-year all-cause mortality (6.8%) and composite adverse events (17.4%) compared to pre-TAVR (20.1% and 30.4%) or concomitant PCI (20.6% and 30.0%).^18^ These findings suggest a potential clinical advantage to deferring PCI, despite known technical challenges related to coronary access after valve implantation.

An observational study utilizing the Nationwide Readmissions Database (2016-2019) evaluated outcomes based on the timing of PCI in relation to TAVR among 5207 patients. Approximately 27% underwent same-day PCI and TAVR, 41.5% had TAVR within 30 days of PCI, and 31.3% had TAVR more than 30 days after PCI. Compared to same-day procedures, patients who underwent TAVR within 30 days of PCI had significantly higher odds of acute kidney injury (adjusted OR 1.49; 95% CI 1.05-2.10; *P* = .024), non-home discharge (aOR 1.53; 95% CI 1.20-1.96; *P* = .001), and 90-day readmission (aOR 1.35; 95% CI 1.04-1.76; *P* = .026). Those with TAVR performed more than 30 days after PCI also had increased odds of non-home discharge (aOR 1.37; 95% CI 1.04-1.82; *P* = .027), although no significant differences were observed for AKI or readmission in that group.^19^ These findings suggest that separating PCI and TAVR by a longer interval may mitigate certain postprocedural risks, while short-interval staging may carry excess morbidity.

### Meta-analyses

A network meta-analysis published in Clinical Cardiology by Fallahtafti et al provided a comparative synthesis of PCI strategies around TAVR. This analysis suggested higher 30-day mortality associated with concomitant (during TAVR) PCI (RR: 2.46, CI: 1.40-4.32), compared to either pre- or post-TAVR PCI (RR: 1.24, CI: 0.94-1.63; RR: 1.37, CI: 0.97-1.94). However, it also reported higher 1-year mortality in the group receiving PCI after TAVR, likely related to challenges with coronary access and procedural complexity.^20^

Another recent meta-analysis published in the Journal of Clinical Medicine by Caminiti et al analyzed PCI timing specifically in stable CAD populations undergoing TAVR. It concluded that PCI after TAVR was associated with lower mortality compared to PCI performed before TAVR (OR 2.48; 95% CI 1.19-5.20; *P* = .02).^6^ These findings supported a strategy of selective or deferred coronary intervention post-valve implantation, emphasizing patient and anatomical characteristics.

### Summary of Current Evidence

Collectively, these studies demonstrate significant uncertainty regarding optimal PCI timing relative to TAVR, reflecting the complexity of decision-making required in this patient population. While randomized trials have not consistently shown clear benefit for pre-TAVR PCI, observational studies highlight procedural challenges associated with deferring PCI until after valve implantation. Future trials are needed to better define patient-specific strategies and refine existing guidelines.

## Coronary Access After TAVR

Coronary access following TAVR is a critical consideration, particularly when planning PCI after valve deployment. Multiple factors can complicate coronary engagement, and understanding these is essential for interventional planning and device selection.

### Valve Type and Anatomy

- Self-expanding valves (eg, Medtronic Evolut, Boston Scientific ACURATE):
  - A prospective study (RE-ACCESS 2) found unsuccessful coronary cannulation in ~5.5% of cases with self-expanding valves; misalignment of commissural posts notably impacted access, especially with Evolut devices which were strongly associated with failure to cannulate both coronaries (OR 24.7; 95% CI 1.9-312.9; *P* = .01).^5^
  - Coronary access may be particularly challenging for the right coronary artery if stent frame cells overlap the ostium
- Balloon-expandable valves (eg, Edwards Sapien):
  - Generally associated with more reliable coronary access due to shorter frame and open cell design.
  - However, commissural tabs and high implantation risk interference, especially when coronary ostia are low in height.^21,22^

### Commissural Alignment

- Improper alignment of transcatheter valve commissures with native valve anatomy significantly impedes coronary catheter engagement.
- Techniques to optimize commissural alignment, such as standardizing valve rotation and delivery orientation, can improve access after TAVR.^23,24^

### Aortic Root Anatomy and Implant Depth

- Anatomical factors, including coronary height, sinus of Valsalva width, and sinotubular junction geometry, affect post-TAVR coronary access.^25^
- A 30% failure rate for selective coronary angiography has been reported post redo TAVR, with deep or high valve implantation further complicating access.^25-27^

### Catheter Techniques and Tools

- For self-expanding valves, identifying the correct frame cell aligned with coronary ostia via aortography aids catheter selection.
- A supportive strategy often includes using smaller Judkins catheters or specialized devices like the Ikari guide catheter, coronary wires, and guide extensions to facilitate access.^22^
- In difficult anatomy, adjunctive devices such as guidewire “rails” or guide extension catheter balloon-assisted tracking, can be utilized to enhance engagement.^28^
- En face (short-axis) fluoroscopic view can be combined with the standard perpendicular view to improve visualization of valve orientation and facilitate selective coronary cannulation in challenging post-TAVR anatomy.^29^

### Clinical Implications

- The difficulty of PCI access post-TAVR may influence timing decisions, making pre-TAVR PCI more appealing in certain patients with specific anatomy.
- As TAVR-in-TAVR procedures become more common, access concerns will intensify, especially in younger or lower-risk patients.

### Conclusion

Coronary access after TAVR is a multifaceted issue influenced by valve design, implantation technique, patient anatomy, and operator strategy. Awareness of these challenges along with proactive techniques like commissural alignment and tailored catheter selection, is essential for ensuring safe and effective post-TAVR revascularization.

The decision to perform PCI before, during, or after TAVR involves trade-offs that must balance anatomical, procedural, and patient-related factors. Table 2 summarizes the advantages and disadvantages of each strategy based on current literature and practice patterns.

**Table 2.** Comparison of the Advantages and Limitations of Percutaneous Coronary Intervention (PCI) Performed Before, During, or After Transcatheter Aortic Valve Replacement (TAVR).

Timing	Pros	Cons
Pre-TAVR PCI	- Simplifies coronary access (native anatomy) - Avoids interference from prosthetic valve - May reduce ischemic risk during TAVR	- Requires staged procedure - May delay TAVR - Increased bleeding risk in short succession
Peri-TAVR PCI	- Single hospitalization - Efficient workflow - May be logistically easier	- Limited by access difficulty during TAVR - Higher procedural complexity - Longer procedural time
Post-TAVR PCI	- Defers revascularization if lesions aren’t ischemia-driving - Avoids overtreatment - Safer in frail patients	- Valve frame may obstruct coronary access - May require complex catheter techniques - Increased risk if urgent PCI needed

Each approach presents unique procedural and clinical trade-offs related to coronary access, timing, and patient safety considerations.

## Special Considerations

When planning PCI timing relative to TAVR, several patient- and lesion-specific factors require special consideration due to their potential impact on clinical outcomes, procedural complexity, and peri-procedural risks.

### High SYNTAX Score Lesions

Patients with a high SYNTAX score (⩾23) present particular challenges due to increased lesion complexity and procedural risk. High SYNTAX scores are associated with worse clinical outcomes following PCI, including higher rates of procedural complications, restenosis, and subsequent coronary revascularization.^30,31^ For these patients, coronary artery bypass grafting (CABG) remains the gold standard; however, many TAVR candidates may not be suitable surgical candidates due to advanced age or comorbidities.^9^ PCI in high-SYNTAX score lesions before TAVR may increase bleeding and ischemic complications, prompting careful evaluation by a multidisciplinary heart team.^32,33^

### Left Main or Proximal LAD Lesions

The presence of left main (LM) or proximal left anterior descending artery (LAD) lesions is a major consideration in patients undergoing TAVR. These lesions, if untreated, carry a substantial risk of myocardial ischemia during valve deployment and hemodynamic instability post-procedure. Most centers advocate for addressing significant LM or proximal LAD lesions with PCI before TAVR, given the higher stakes of peri-procedural ischemia in these territories.^11^ Nonetheless, increased procedural complexity and bleeding risks associated with PCI should be considered carefully in elderly or frail patients.

### Frailty and Comorbidities

Frailty significantly impacts procedural risk and clinical outcomes in TAVR patients. Frail patients experience higher rates of peri-procedural complications, longer recovery times, and increased bleeding events with invasive interventions like PCI. For frail patients, a more conservative, staged approach, potentially performing TAVR first and deferring PCI unless clinically necessary, may be prudent.^34,35^

Additionally, comorbidities, especially chronic kidney disease (CKD) and advanced heart failure, further complicate decision-making. CKD patients have an elevated risk of contrast-induced nephropathy, particularly when PCI and TAVR are performed in close succession. Minimizing contrast volume and spacing out PCI and TAVR procedures can reduce the risk of renal complications.^36,37^

### Antithrombotic and Bleeding Risk

Management of antiplatelet and anticoagulant therapy is a major challenge. Dual antiplatelet therapy (DAPT), standard after PCI, significantly increased bleeding risks when performed close to TAVR. Balancing bleeding risk with ischemic protection requires careful considerations of patient-specific bleeding risk factors, potentially favoring PCI before TAVR to simplify peri-procedural anticoagulation management.^38,39^

In patients requiring oral anticoagulation (eg, those with atrial fibrillation), bleeding risks further escalate, necessitating tailored, individualized approaches often guided by collaborative heart team assessments.^40^

### Conclusion

**Figure 1.**
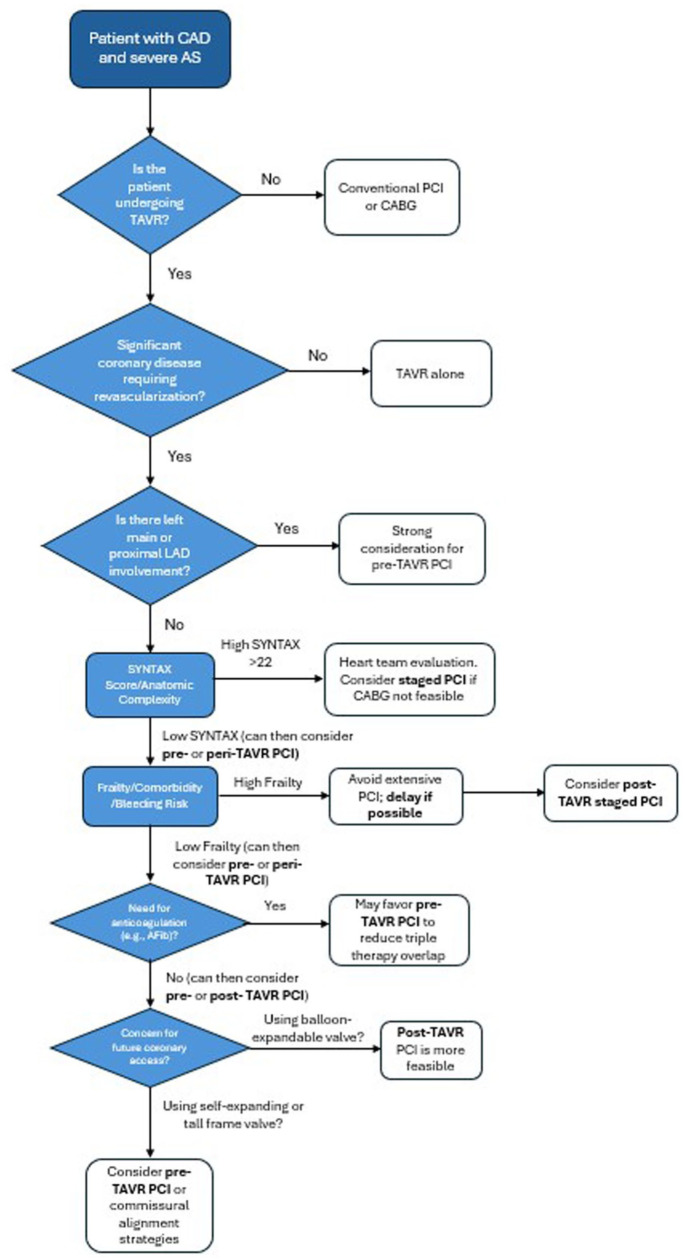
Proposed Decision Algorithm for PCI Timing in Patients Undergoing TAVR. This flowchart presents a proposed clinical algorithm to guide the timing of percutaneous coronary intervention in patients with severe aortic stenosis undergoing transcatheter aortic valve replacement. The decision tree incorporates anatomical complexity (eg, SYNTAX score), lesion location, comorbidities, bleeding risk, need for anticoagulation, and anticipated coronary access challenges to help determine whether PCI should be performed pre-, peri-, or post-TAVR.

Special considerations such as complex coronary anatomy, left main involvement, frailty, comorbidities, and anticoagulation management are critical in determining optimal PCI timing in TAVR candidates. A heart-team-based, individualized assessment remains paramount, incorporating patient preferences, anatomical complexities, and careful risk-benefit analyses to guide clinical decisions. Based on the synthesis of current evidence and expert consensus, we propose a clinical decision algorithm to guide individualized PCI timing in patients undergoing TAVR, integrating anatomical complexity, lesion location, frailty, and procedural risks (Figure 1).

## Future Directions

Despite growing literature addressing PCI timing in patients undergoing TAVR, several knowledge gaps remain. Addressing these areas through focused clinical trials, advancements in procedural techniques, and improved diagnostic tools will be essential for optimizing future patient care.

### Ongoing and Planned Clinical Trials

Several prospective studies are currently underway or planned to address existing controversies directly. Trials such as COMPLETE TAVR (ClinicalTrials.gov identifier: NCT04634240) aim to evaluate systematically whether complete revascularization prior to TAVR reduces cardiovascular events compared to TAVR alone.^11,41^ Additionally, the forthcoming outcomes from extended follow up of the NOTION-3 trial and large-scale international registries like TAVI-PCI will further clarify the long-term risks and benefits of different PCI timing strategies.^17,42^

### Personalized Decision-Making and Risk Scores

Current PCI timing strategies predominantly rely on clinical judgment and anatomical evaluation. Future approaches must integrate more precise patient risk prediction tools and personalized risk scores incorporating factors like frailty, comorbidities, and coronary lesion complexity, and anatomical predictors of difficult coronary access.^34,35^ Advanced predictive models using artificial intelligence (AI) and machine learning to stratify risk could guide individualized timing of PCI relative to TAVR.^43,44^

### Innovations in Valve Technology and Delivery Techniques

Next generation transcatheter valves with improved commissural alignment, lower stent-frame profiles, and tailored anatomical adaptability are under development. These innovations aim to minimize future coronary access challenges, potentially expanding safe and practical post-TAVR PCI options.^14,23,24^ Additionally, further refinement of TAVR delivery systems that allow more precise control over valve positioning and orientation could significantly enhance coronary engagement post-implantation.^45,46^

### Hybrid Imaging and Fusion Technologies

Advancements in multimodality imaging, including computed tomography angiography (CTA) fusion with live fluoroscopy, have the potential to greatly improve procedural planning and execution. Integrating advanced imaging technologies can help ensure optimal valve positioning, commissural alignment, and guide selective coronary engagement post-TAVR.^47-49^

### Coronary Physiology and Functional Assessment

The incorporation of functional and physiological assessment of coronary lesions (eg, rational flow reserve [FFR], and instantaneous wave-free ratio [iFR]) prior to TAVR is relatively unexplored. While these modalities may help refine PCI decision-making, it is important to note that the reliability of functional lesion assessment after TAVR is limited, as valve implantation alters aortic root hemodynamics and may affect coronary pressure measurements. Future trials evaluating the role of invasive physiology in guiding PCI decision making around TAVR could substantially enhance the precision of clinical decisions, potentially reducing unnecessary PCI and associated complications.^50-52^

### Long-Term Outcomes and Durability Considerations

With TAVR increasingly employed in younger and lower-risk populations, understanding the long-term implications of PCI timing on valve durability, coronary access, and repeat interventions such as valve-in-valve procedures) will become increasingly critical. Studies focusing specifically on younger cohorts and longer-term follow up (beyond 5 years) are necessary.^53-55^

### Conclusion

Future directions should focus on prospective, randomized trials addressing current evidence gaps, technology advancements to facilitate coronary access post-TAVR, precision medicine approaches for individualized decision-making, and long-term patient follow-up to inform evolving practice guidelines and optimize clinical outcomes.

## Conclusion

Determining the optimal timing of PCI in patients undergoing TAVR remains challenging due to the complexity of clinical presentations, anatomical variability, and mixed clinical outcomes observed across different timing strategies. Current evidence from randomized trials, such as ACTIVATION and NOTION-3, as well as numerous observational studies and meta-analyses, underscore the absence of a universally superior approach, highlighting the necessity of individualized decision-making. Factors including coronary lesion complexity, anticipated procedural difficulty, frailty, renal function, and antithrombotic therapy management must be carefully considered by multidisciplinary heart teams. Ongoing research, technological innovations in valve and catheter design, improved imaging and physiological assessment, and personalized risk stratification tools will further refine our approach to PCI timing in this growing patient population. As TAVR continues to expand into younger, lower-risk cohorts, addressing current gaps through rigorous research will be paramount to optimize patient outcome and procedural safety.
